# AHR2 Mutant Reveals Functional Diversity of Aryl Hydrocarbon Receptors in Zebrafish

**DOI:** 10.1371/journal.pone.0029346

**Published:** 2012-01-05

**Authors:** Britton C. Goodale, Jane K. La Du, William H. Bisson, Derek B. Janszen, Katrina M. Waters, Robert L. Tanguay

**Affiliations:** 1 Department of Environmental and Molecular Toxicology, Environmental Health Sciences Center, Oregon State University, Corvallis, Oregon, United States of America; 2 Pharmaceutical Biochemistry Group, School of Pharmaceutical Sciences, University of Geneva, Geneva, Switzerland; 3 Computational Biology and Bioinformatics Group, Pacific Northwest National Laboratory, Richland, Washington, United States of America; Istituto Dermopatico dell'Immacolata, Italy

## Abstract

The aryl hydrocarbon receptor (AHR) is well known for mediating the toxic effects of TCDD and has been a subject of intense research for over 30 years. Current investigations continue to uncover its endogenous and regulatory roles in a wide variety of cellular and molecular signaling processes. A zebrafish line with a mutation in *ahr2* (*ahr2* ^hu3335^), encoding the AHR paralogue responsible for mediating TCDD toxicity in zebrafish, was developed via Targeting Induced Local Lesions IN Genomes (TILLING) and predicted to express a non-functional AHR2 protein. We characterized AHR activity in the mutant line using TCDD and leflunomide as toxicological probes to investigate function, ligand binding and CYP1A induction patterns of paralogues AHR2, AHR1A and AHR1B. By evaluating TCDD-induced developmental toxicity, mRNA expression changes and CYP1A protein in the AHR2 mutant line, we determined that *ahr2* ^hu3335^ zebrafish are functionally null. *In silico* modeling predicted differential binding of TCDD and leflunomide to the AHR paralogues. AHR1A is considered a non-functional pseudogene as it does not bind TCCD or mediate *in vivo* TCDD toxicity. Homology modeling, however, predicted a ligand binding conformation of AHR1A with leflunomide. AHR1A-dependent CYP1A immunohistochemical expression in the liver provided *in vivo* confirmation of the *in silico* docking studies. The *ahr2* ^hu3335^ functional knockout line expands the experimental power of zebrafish to unravel the role of the AHR during development, as well as highlights potential activity of the other AHR paralogues in ligand-specific toxicological responses.

## Introduction

The aryl hydrocarbon receptor (AHR), while best known for its role as an environmental sensor and mediator of 2,3,7,8-tetrachlorodibenzo-*p*-dioxin (TCDD) toxicity, has captured attention in recent years with a growing body of research elucidating its endogenous functions. As a member of the bHLH-Per-Arnt-Sim(PAS) family of proteins, the AHR is a transcriptional regulator containing two evolutionarily-conserved domains: a basic helix-loop-helix (bHLH) domain, which enables binding to aromatic hydrocarbon-responsive elements (AHREs), and a PAS domain, consisting of two 51- amino acid imperfect repeats (PAS-A and PAS-B), responsible for dimerization, ligand binding and interactions with other proteins [1], [2]. Originally discovered for its role in modulating TCDD sensitivity in mice, the AHR binds a wide variety of ligand structures, including polycyclic and halogenated aromatic hydrocarbons (PAH and HAHs). Ligand binding induces disassociation from a cytoplasmic protein complex and translocation to the nucleus where the AHR heterodimerizes with the aryl hydrocarbon nuclear translocator (ARNT) [3], [4], [5]. The AHR-ARNT heterodimer, along with other transcriptional enhancers, binds to AHREs and activates transcription of CYP1A, as well as NQO1, ALDH3A1, UGT1A6 and many other genes involved in metabolism, oxidative stress response and cell signaling [6], [7]. The role of the AHR in mediating toxicity of environmental contaminant exposure has been extensively studied (reviewed in [8], [9], [10]), and mechanism of action in immune, reproductive, developmental and other toxicological responses remain active areas of investigation. The diversity of physiological systems impacted by AHR activation and its crosstalk with other regulatory pathways support the notion that endogenous functions for the receptor likely preceded its role as an environmental sensor [11].

TCDD binding activity of the AHR is conserved among vertebrates. Substitutions in critical residues produce variation in ligand affinity, which underlies differences in TCDD sensitivity between species, inbred mouse strains, and wild fish populations [4], [12], [13], [14]. Structural comparisons of receptors provide information necessary for risk assessment extrapolation between species, as well as insight into receptor evolution [15]. In addition, *in silico* modeling of the AHR has emerged as a powerful screening tool for identifying novel AHR ligands [16], [17].

Developing fish embryos are extremely sensitive to AHR-mediated planar hydrocarbon toxicity and hold a number of experimental advantages including development external to the mother, ease of observation, and genetic tractability. As such, zebrafish are a valuable model for investigation of developmental signaling processes in the context of xenobiotic exposures [18], [19]. In teleosts, genome-wide duplication events have resulted in co-orthologs for many mammalian genes. While some gene duplicates have become non-functional, others have been evolutionarily conserved via the partitioning of functions between paralogues [20]. Three AHR isoforms have been identified in zebrafish: AHR1A, AHR1B, and AHR2 [12], [21], [22], [23]. Numerous studies with known AHR ligands, however, have identified AHR2 as the primary mediator of early life stage toxicological effects in zebrafish [24], [25], [26]. Antisense oligonucleotide (morpholino) knockdown of AHR2 affords almost complete resistance to TCDD-induced developmental toxicity, and prevents the inhibitory effects of AHR ligands on epimorphic regeneration [25], [27]. Toxicity of many other HAHs and PAHs is also primarily dependent on AHR2. While AHR1B does bind TCDD, it is less sensitive to activation by TCDD than AHR2 [23], [24], [28]. In contrast, AHR1A does not bind TCDD and is deficient in transactivation activity [21], [23]. Beyond functioning as xenobiotic sensors, the zebrafish AHR paralogues are proposed to serve endogenous functions that have yet to be elucidated.

Recent studies have highlighted endogenous roles for the AHR in a complex array of immune system, cell cycle regulatory, reproductive and developmental processes [10], [29], [30], [31], [32]. AHR knockout mouse strains developed by three different groups illustrate the importance of the AHR in normal liver development and immune function, and continue to expand understanding of the receptor's role in both toxicological responses and normal physiology [33], [34], [35]. A functional zebrafish AHR2 knockout line will allow for investigation of the biological functions of the receptor throughout the zebrafish lifespan, and will eliminate the concern of incomplete knockdown that can occur with morpholinos. Complete loss of AHR2 activity in a zebrafish line will also enable functional analysis of the other two receptors, which has to date been experimentally difficult. As the primary mediator of TCDD toxicity, we proposed AHR2 as a target of great value to the zebrafish community for Targeting Induced Local Lesions IN Genomes (TILLING). Here we describe characterization of AHR function in the first TILLING-indentified AHR2 mutant zebrafish. We report loss of AHR2 function in a mutant AHR2 line, and present evidence of ligand- and tissue-specific activation and function of AHR1A and AHR1B.

## Results

### Generation of a functionally null AHR2 zebrafish line

**Figure 1:**
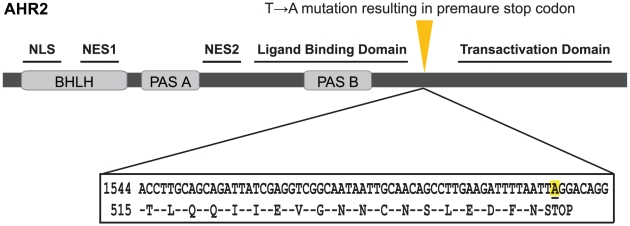
Schematic diagram of predicted AHR2 protein in *ahr2* ^hu3335^ zebrafish. The *ahr2* ^hu3335^ zebrafish line has a T to A point mutation in residue 534, resulting in a premature stop codon in the transactivation domain of the protein. The predicted truncated protein contains the ligand binding, DNA binding and ARNT binding domains, but lacks the transactivation domain previously shown to be essential for a functional AHR2 protein [21], [58]. NLS: nuclear localization signal, NES1: nuclear export signal 1, NES2: nuclear export signal 2.

The *ahr2* ^hu3335^ line was established, upon request, by the Hubrecht institute from a TILLING-identified founder with a TTG to TAG point mutation in residue Leu534, resulting in a premature stop codon in the transactivation domain of AHR2 (Figure 1). While the bHLH and PAS domains are predicted to remain intact in the truncated protein, the transactivation domain of zebrafish AHR2 is required for transcriptional activation [21]. In addition, the premature stop codon location is >55 nucleotides upstream of an exon-exon boundary, likely rendering the mutant AHR2 mRNA a target of nonsense-mediated mRNA decay, which will be further discussed below [36].

**Figure 2:**
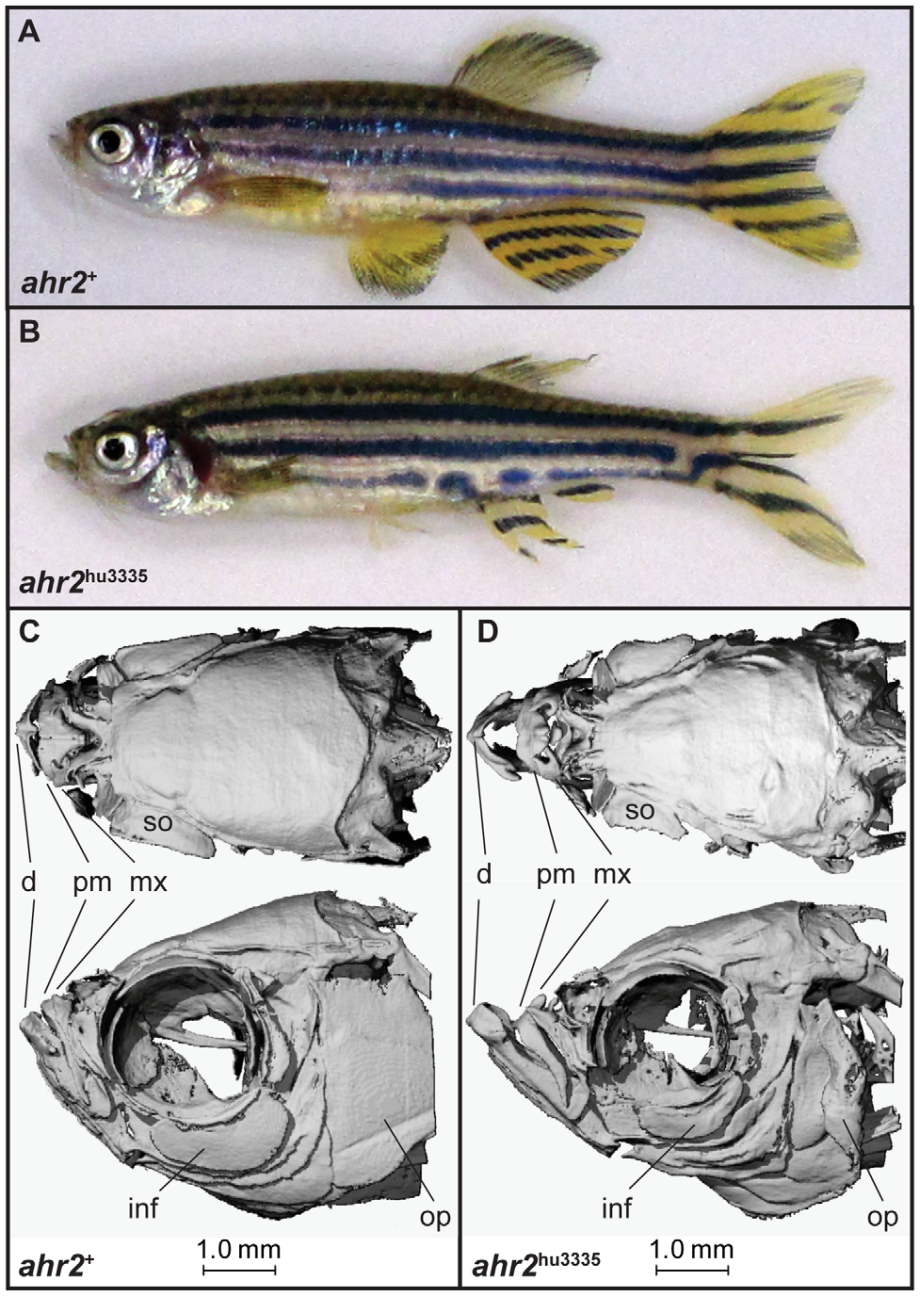
Fin and skeletal abnormalities observed in adult *ahr2* ^hu3335^ zebrafish. **A–B**) Brightfield and (**C–D**) microCt imaging of adult *ahr2* ^+^ and *ahr2* ^hu3335^zebrafish. Notable differences were observed in the dentate (d), premaxilla (pm), maxilla (mx), supraorbital (so), infraorbital 3(inf) and operculum (op).

*ahr2* ^hu3335^ zebrafish survived to adulthood with no consistently observed abnormalities during development. Jaw, gill and fin malformations were observed in adult fish, but did not appear to cause significant morbidity or mortality. The fins of *ahr2* ^hu3335^ adult zebrafish are damaged compared to their *ahr2* ^+^ clutch mates, a characteristic which persisted in the offspring of wild-type 5D-outcrossed *ahr2* ^hu3335/+^ zebrafish (Figure 2A,B). Visible jaw malformations in *ahr2* ^hu3335^ adults prompted us to investigate bone structure using non-destructive microCt scanning. MicroCt imaging revealed structural differences in the neurocrania of an *ahr2* ^hu3335^ and an aged-matched wild-type strain 5D adult zebrafish, including a striking extension of the ethmoid and mandibular regions (Figure 2C,D) [37]. Further, the dentary, maxilla and premaxilla of the *ahr2* ^hu3335^ zebrafish had notably different structure, creating an extended mandible. Other bones, such as the orbitals and supraorbitals, appeared smaller in the *ahr2* ^hu3335^ zebrafish, which may be an artifact of scanning reduced bone thickness compared to the wild type [37].

In comparison to their *ahr2* ^+^ and *ahr2* ^+/hu3335^ siblings, spawning activity of *ahr2* ^hu3335^ homozygous crosses was less robust and egg fertilization rates were low (50–75%). As is discussed further in regard to developmental toxicity assays, pericardial edema and jaw malformations occurred with higher incidence in some of the *ahr2* ^hu3335^ clutches. Sporadic spawning activity of *ahr2* ^hu3335^ homozygous crosses and successful in vitro fertilization demonstrated that the *ahr2* ^hu3335^ mutation does not prevent reproductive function in this line. Irregular spawning, however, suggests deficits in reproductive physiology or behavior.

### *ahr2* ^hu3335^ embryos are resistant to TCDD-induced developmental toxicity

**Figure 3:**
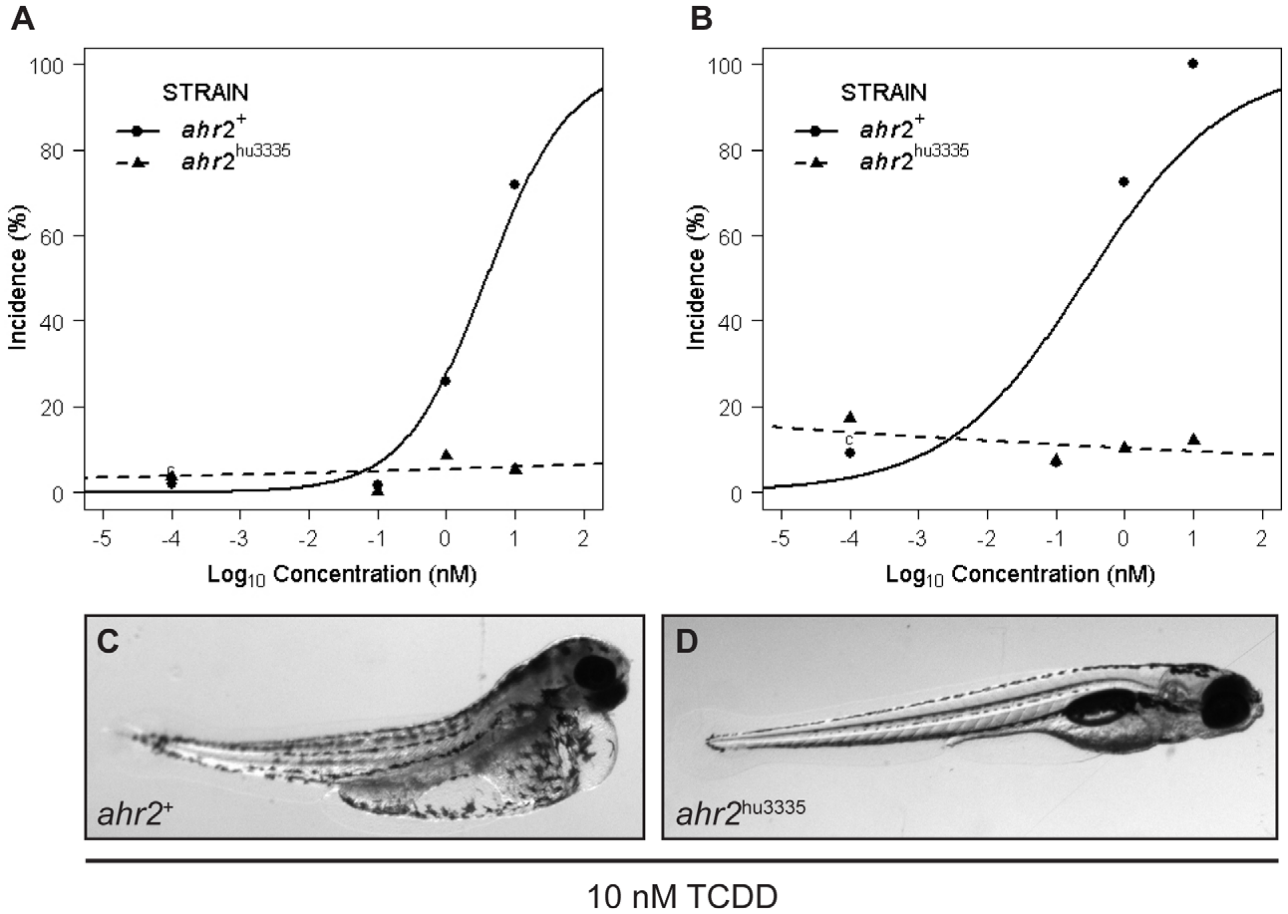
*ahr2* ^hu3335^ embryos are resistant to TCDD-induced developmental abnormalities. **A**) Percent of embryos with axis malformations and **B**) percent incidence pericardial edema at 120 hpf in embryos treated with 0, 0.1, 1 or 10 nM TCDD from 6–24 hpf. Vehicle control groups (c, 0.1% DMSO) are displayed at 10^−4^ for graphing purposes. Data represent three independent experiments with 20 embryos per treatment group. **C**) Representative image of 120 hpf *ahr2* ^+^ and (**D**) *ahr2* ^hu3335^ embryos developmentally exposed to 10 nM TCDD.

To assess AHR2 function in the *ahr2* ^hu3335^ strain, we compared developmental toxicity of TCDD in the *ahr2* ^hu3335^ mutants to *ahr2* ^+^ embryos. Exposure to 0.1, 1 or 10 nM TCDD resulted in a concentration-dependent increase in axis malformations and pericardial edema observed at 120 hpf in the *ahr2* ^+^ embryos (Figure 3A,C). Of the fifteen endpoints evaluated, TCDD concentration was significantly correlated with increases in yolk sac and pericardial edemas, and axis, eye, snout, jaw and trunk malformations (Table 1). Mortality, touch response, fin, pigment, brain, circulatory, somite and otic malformations were not significant responses in either fish line. *ahr2* ^hu3335^ embryos were resistant to TCDD-dependent malformations, and the responses of *ahr2* ^+^ and *ahr2* ^hu3335^ embryos to TCDD exposure were significantly different from each other (Figure 3A,B,D, Table 1). Background pericardial edema and jaw malformations were observed in *ahr2* ^hu3335^ embryos but were not TCDD-dependent.

**Table 1:** Concentration responses for developmental effects observed in TCDD-exposed *ahr2* ^+^ and *ahr2* ^hu3335^ embryos.

Effect	p-value of *ahr2* ^+^ TCDD concentration-response	p-value of *ahr2* ^hu3335^ TCDD concentration-response	p-value of *ahr2* ^hu3335^ and *ahr2* ^+^ differential response to TCDD
**yolk sac edema**	<0.0001	0.7181	0.0004
**Axis**	<0.0001	0.2754	0.0006
**Eye**	<0.0001	1.0000	0.0005
**Snout**	<0.0001	0.6706	0.0004
**Jaw**	<0.0001	0.8632	0.0011
**pericardial edema**	<0.0001	0.0848	0.0002

### mRNA expression indicates the *ahr2* ^hu3335^ mutation abrogates AHR2 function

**Figure 4:**
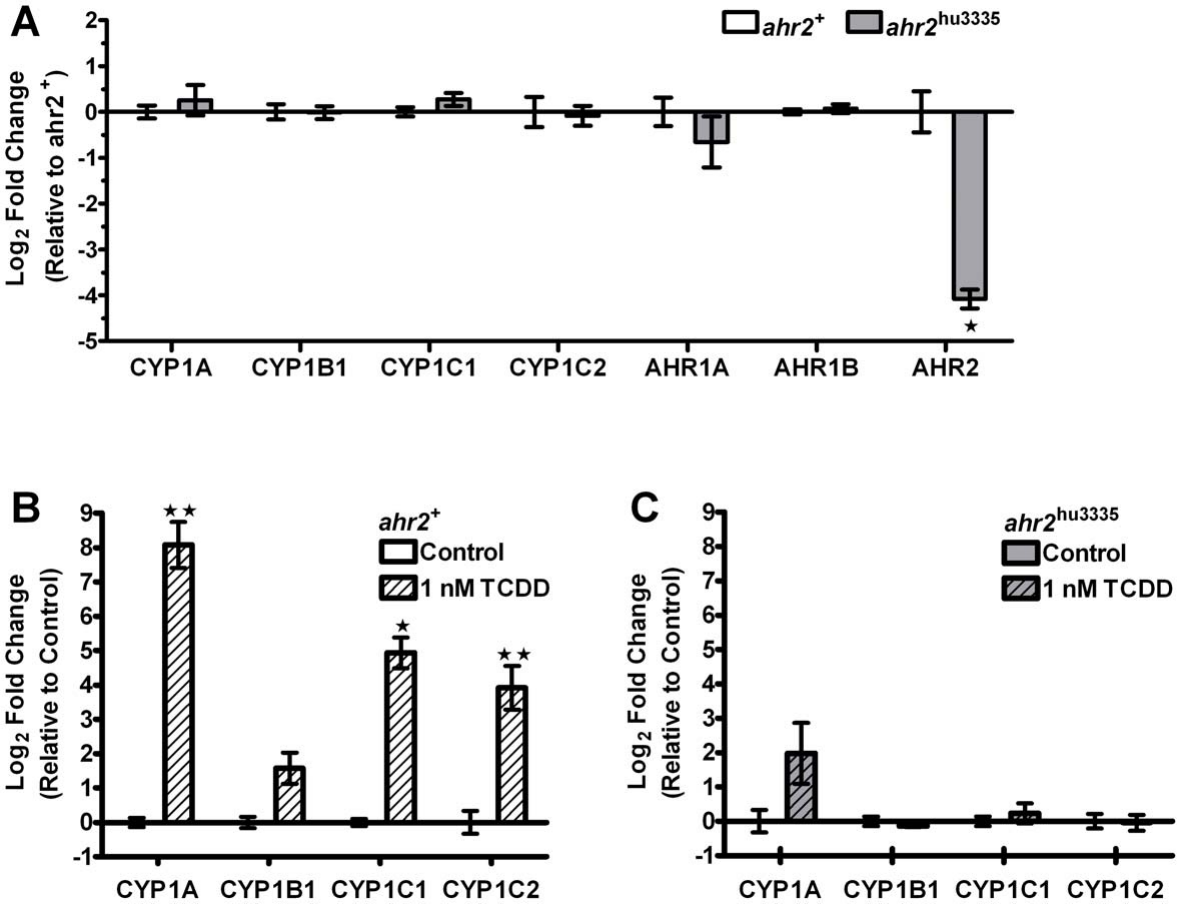
*ahr2* ^hu3335^ embryos express reduced endogenous AHR2 mRNA and are resistant to TCDD-induced CYP induction. **A**) Comparative analysis of AHR1A, AHR1B, CYP1A, CYP1B1, CYP1C1 and CYP1C2 mRNA expression in wild-type 5D and *ahr2* ^hu3335^ mutant embryos at 48hpf . ΔΔCt values were calculated by comparing sample ΔCt values (normalized to β-actin) to the mean *ahr2* ^+^ ΔCt for each gene. Data were analyzed by paired student's t-test, * p<.05. **B**) Developmental exposure (6–24 hpf) to 1 nM TCDD induced significant CYP1A, CYP1C1 and CYP1C2 expression at 48 hpf in *ahr2* ^+^ embryos. Data is shown normalized to vehicle-treated controls and was analyzed with paired student's t-test, *p<.05, ** p<.01. **C**) Developmental exposure to 1 nM TCDD did not induce significant mRNA expression changes in *ahr2* ^hu3335^ embryos. While CYP1A was elevated, the difference was not significant.

We evaluated mRNA expression to further assess AHR2 function, and observed a 16-fold difference in AHR2 transcript abundance between *ahr2* ^+^ and *ahr2* ^hu3335^ embryos (Figure 4A). This supports the hypothesis that AHR2 mRNA is degraded in the *ahr2* ^hu3335^ line. We next examined AHR2-dependent gene expression to determine whether the point mutation perturbs expression of downstream transcriptional targets. Expression of CYP1A, CYP1B1, CYP1C1, CYP1C2, AHR1A and AHR1B transcripts were not significantly different between *ahr2* ^hu3335^ and *ahr2* ^+^ embryos (Figure 4A).

To further confirm the lack of AHR2 functionality, we investigated mRNA expression changes in response to TCDD, which induces AHR2-dependent expression of a number of mRNAs at 48 hpf [38]. Developmental TCDD exposure induced robust expression of CYP1A, CYP1C1 and CYP1C2 mRNA at 48 hpf in *ahr2* ^+^ embryos relative to vehicle-treated controls (Figure 4B). As expected in the absence of a functional AHR2, mRNA expression was not significantly elevated in *ahr2* ^hu3335^ embryos exposed to TCDD (Figure 4C).

### AHR1A is predicted to bind leflunomide but not TCDD

**Figure 5:**
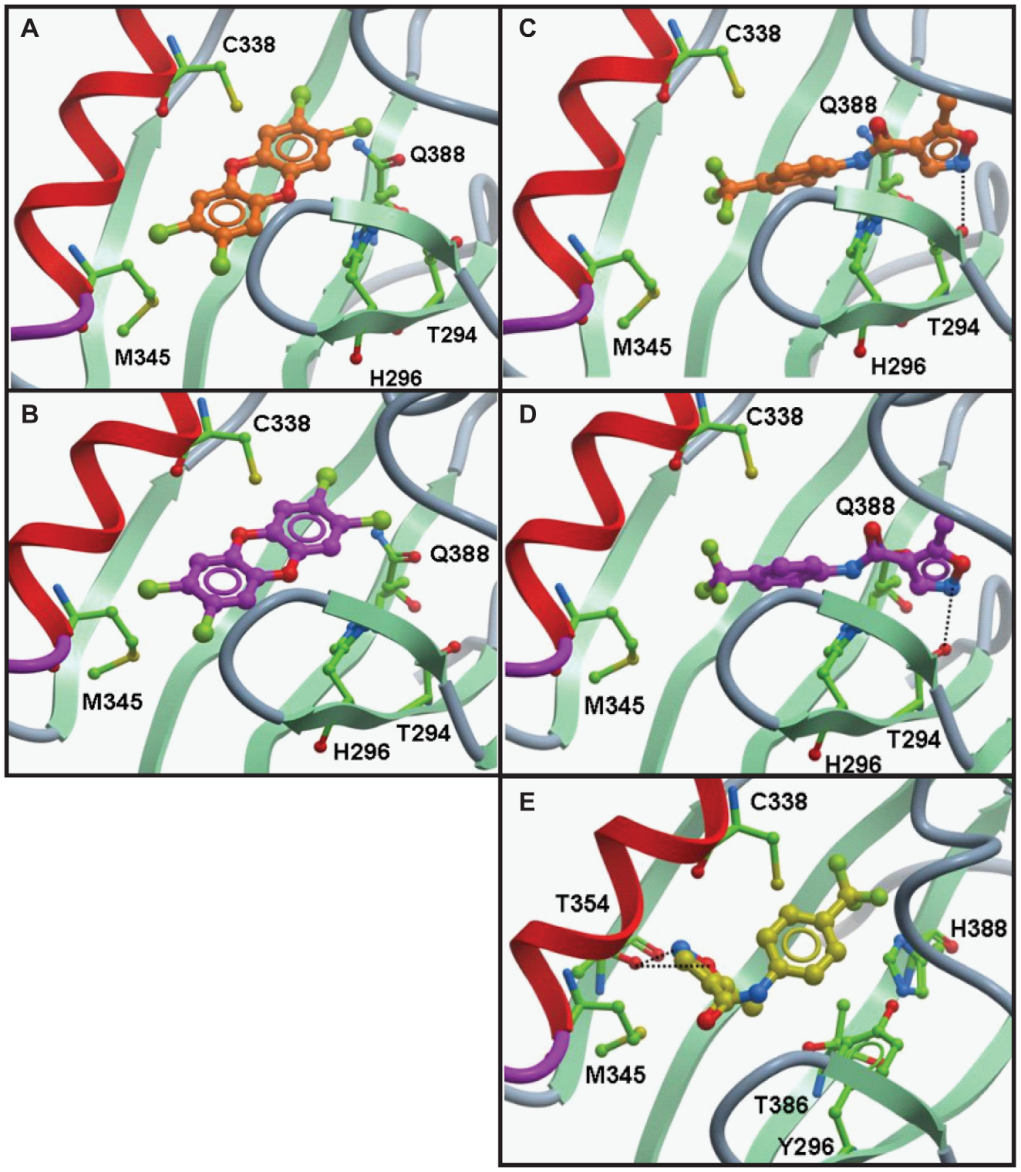
Molecular docking of TCDD and Leflunomide in zebrafish AHR isoforms. **A**) TCDD docking orientation in zebrafish AHR2- and **B**) AHR1B-LBD homology model binding pocket (ICM v3.5-1n, Molsoft). **C**) Leflunomide docking orientation into AHR2-, **D**) AHR1B- and **E**) AHR1A homology model binding pockets. The residues are displayed as sticks and colored by atom type with the carbon atoms in green. The protein backbone is displayed as ribbon and colored by secondary structure. The ligand is displayed as sticks and colored by atom type with carbon atoms in orange (**A, C**), magenta (**B, D**) and yellow (**E**). H-bonds are represented by black dashed lines.

We recently reported a homology model that has been used to predict binding affinity of potential ligands to the human, mouse and zebrafish AHRs [39]. In order to investigate differential function of the zebrafish AHR paralogues, we tested TCDD and a known AHR ligand with a non-classical structure, leflunomide, in a series of molecular docking studies. Sequence alignment of the mouse and zebrafish AHR-PASB domains produced identities of 65.1% (zfAHR1A), 78.5% (zfAHR1B) and 70.5% (zfAHR2). High similarity between the three isoforms at the primary and predicted tertiary structural levels was also noted, with 74.3% (AHR2/1B) and 69.9% (AHR1B/1A) identity. TCDD and leflunomide were docked into zebrafish AHR1A-, AHR1B-, and AHR2-LBD homology models. TCDD docked in AHR2 and AHR1B with predicted binding energies of −3.97 kcal/mol and −4.86 kcal/mol, respectively, but was unable to dock in AHR1A (Table 2, Figure 5A,B). Leflunomide was also able dock in AHR2 and AHR1B, with predicted binding energies of −2.13 kcal/mol and −1.97 kcal/mol, respectively (Table 2, Figure 5C,D). Interestingly, in contrast to TCDD, leflunomide docked into AHR1A, but in a unique orientation [17] (Figure 5E).

**Table 2:** Predicted binding energy values for zebrafish AHR2, AHR1B and AHR1A (kcal/mol).

	AHR2	AHR1B	AHR1A
**TCDD**	−3.97	−4.86	ND
**Leflunomide**	−2.13	−1.97	−2.19

ND – unable to dock.

AHR1A possesses specific residues that play potential roles in TCDD insensitivity [23]. Key residues characterized in the mouse AHR-LBD influencing TCDD binding are conserved in zebrafish AHR2 and AHR1B, which are both TCDD sensitive [17], [40], [41]. In AHR1A, residues His296, Ala386 and Gln388 have been substituted with Tyr296, Thr386 and His388 [23]. The side chains of these residues cause both decreased volume and altered polarity of the AHR1A binding pocket, in comparison to AHR2, AHR1B, as well as mouse and human AHRs [17]. TCDD docking is consequently not possible in AHR1A, which has been confirmed both *in vitro* and *in vivo* [21], [23]. Homology modeling predicted that leflunomide, however, is able to dock in AHR1A with a unique orientation not found in human, mouse, or zebrafish AHR1B and AHR2 isoforms [39]. As shown previously, leflunomide docks in AHR2 and AHR1B with a hydrogen bond (HB) interaction between the nitrogen atom of the isoxazole ring of the ligand and the OH of the side chain of Thr294 ([39], Figure 5C,D). Here we employed the homology model to examine AHR1A interaction with leflunomide for the first time, and discovered that the leflunomide docking position is flipped and a double HB interaction between the nitrogen and oxygen of the isoxazole ring of the ligand and the side chain of Thr354 is formed (Figure 5E). A binding energy of −2.19 kcal/mol was predicted which is in the range calculated for the other two isoforms (Table 2). Based on these data, we predicted that leflunomide would be a functional AHR1A ligand.

### CYP1A protein induction patterns are ligand- and AHR isoform-dependent

**Figure 6:**
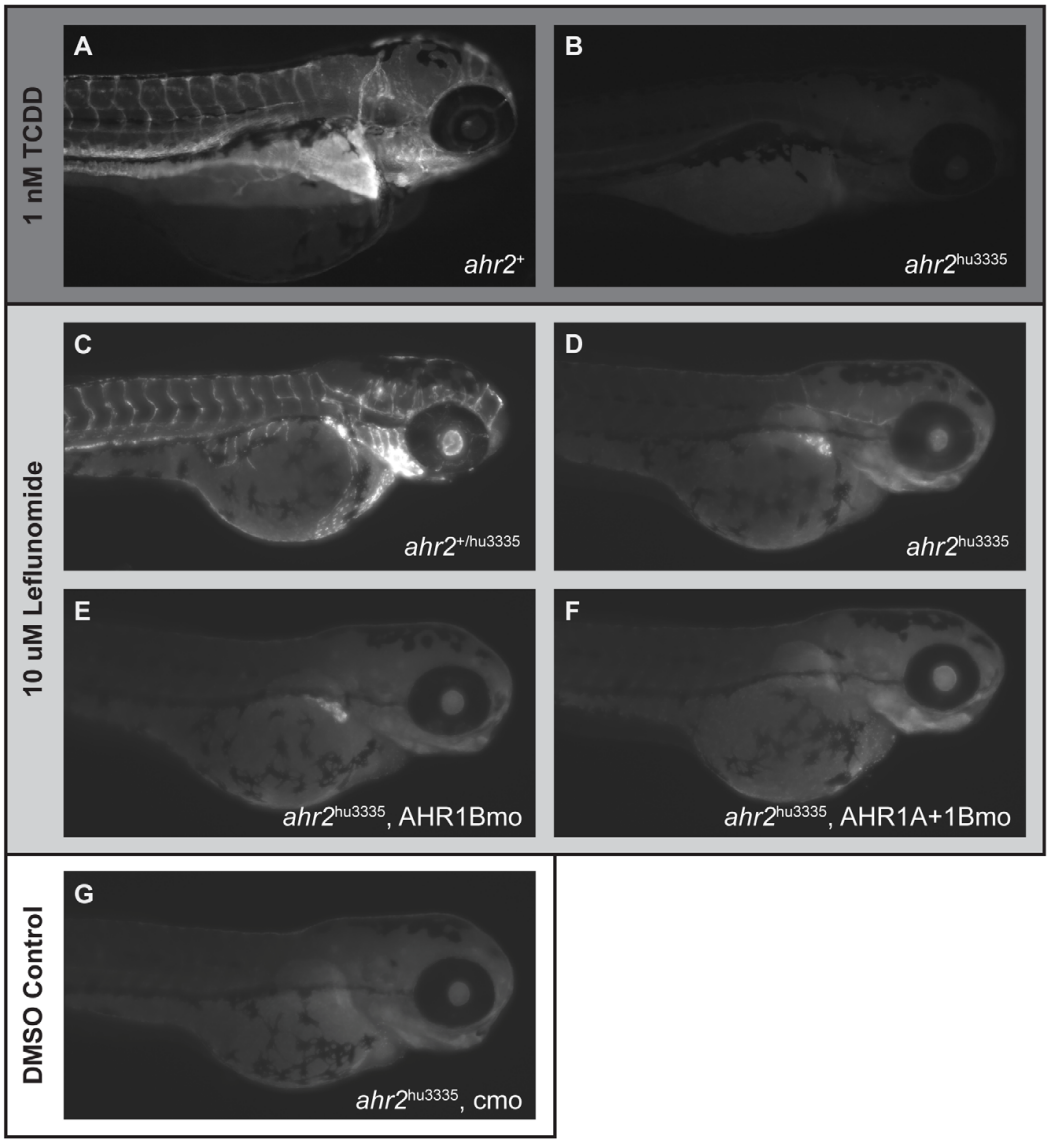
CYP1A protein expression patterns are ligand- and AHR isoform-dependent. CYP1A expression at 120 hpf in (**A**) *ahr2* ^+^ and (**B**) *ahr2* ^hu3335^ larvae following exposure to 1 nM TCDD from 6–24 hpf. **C**) Leflunomide-induced CYP1A expression at 72 hpf in wild-type and (**D**) *ahr2* ^hu3335^ mutants. **E**) Leflunomide-induced CYP1A expression in AHR1B-morphant *ahr2* ^hu3335^ larvae and **F**) *ahr2* ^hu3335^ larvae co-injected with AHR1A and AHR1B morpholinos. (**G**) DMSO control. TCDD-exposed embryos were IHC processed side-by-side and imaged at 120 hpf using the same exposure settings and a single focal plane. Leflunomide-exposed embryos and DMSO control were processed side-by-side and imaged at 72 hpf using the same exposure times; images were created from a z-stack of 10 15.4 uM slices centered on the liver.

We used immunohistochemical analysis of CYP1A protein expression as a biomarker of AHR activation to investigate *in vivo* AHR ligand binding patterns in TCDD and leflunomide-exposed larvae. Exposure to 1 nM TCDD from 6–24 hpf induces AHR2-dependent CYP1A expression in a number of tissues, including the heart, liver and enteric tract, with the predominant expression in the vascular endothelium of larvae (Figure 6A) [42]. We focused our evaluation of AHR function on the most robust CYP1A induction patterns, which were observed in vasculature and liver [42], [43]. As predicted by the qRT-PCR results, CYP1A protein expression in TCDD-exposed *ahr2* ^hu3335^ larvae was limited to faint vascular expression, just above background, in all embryos examined (Figure 6B). Exposure to 10 nM TCDD, which induced severe malformations and robust CYP1A expression in wild-type embryos, did not notably increase CYP1A expression in *ahr2* ^hu3335^ larvae (data not shown).

To confirm the predicted binding of leflunomide to all three zebrafish AHRs *in vivo*, we examined CYP1A induction in *ahr2* ^hu3335^ larvae exposed to 10 µM leflunomide from 48–72 hpf. In comparison to wild type larvae, vascular CYP1A expression was drastically reduced in leflunomide-exposed *ahr2* ^hu3335^ larvae (Figure 6C, D). In contrast to TCDD exposure, however, AHR2-independent CYP1A expression was observed in the developing livers of leflunomide-exposed *ahr2* ^hu3335^ larvae (Figure 6D). This expression pattern persisted in larvae exposed until 120 hpf, with vascular expression remaining low and liver expression increasing, likely due to growth that occurs from 72–120 hpf (data not shown).

Based on molecular docking studies, we hypothesized that leflunomide induced CYP1A in *ahr2* ^hu3335^ larvae via activation of the other AHR homologs, and utilized splice-blocking morpholinos to transiently knock down AHR1A and AHR1B separately and in combination. We conducted immunohistochemical analysis of CYP1A expression at 72 hpf to capture the window of morpholino efficacy, which was confirmed with PCR using primers flanking the target sites (Figure S1). As the liver is small at 72 hpf, we employed double-staining with a hepatocyte nuclear factor 4α (HNF4α) antibody to confirm the presence of hepatocytes [44] (data not shown). CYP1A expression in AHR1B morpholino-injected *ahr2* ^hu3335^ larvae persisted in the liver (Figure 6E), but was notably absent in the vasculature. In contrast, injection of the AHR1A morpholino in *ahr2* ^hu3335^ embryos blocked leflunomide-induced expression of CYP1A in the liver, while faint vascular expression remained. When co-injected, the AHR1A and AHR1B morpholinos blocked all CYP1A expression in leflunomide-exposed *ahr2* ^hu3335^ larvae (Figure 6F). When expression of all 3 AHR isoforms was eliminated, CYP1A expression in leflunomide-exposed embryos was indistinguishable from vehicle-exposed controls (Figure 6G).

## Discussion

*ahr2* ^hu3335^ zebrafish, homozygous for a point mutation in *ahr2*, survive to adulthood and are functional AHR2 knockouts by all measures tested. The premature stop codon in residue 534 is predicted to result in a non-functional protein due to its truncated transactivation domain. Though we cannot exclude the possibility that some biological activity of a potential cryptic protein remains, we saw no evidence to support its presence. Analysis of *ahr2* ^hu3335^ mRNA levels suggests that the mutant AHR2 transcript is at least partially degraded and the truncated protein may be present only at very low levels, if at all.

The *ahr2* ^hu3335^ adult zebrafish exhibit notable fin and skeletal differences compared to wild type. We also observed a higher background of developmental abnormalities in *ahr2* ^hu3335^ larvae. These phenotypes may not necessarily be due to the mutation; reduced spawning and small clutch sizes of *ahr2* ^hu3335^ zebrafish limited the choice of embryos for experiments, whereas large wild type clutches allow for precise selection of high-quality embryos. Studies in both AHR-deficient and AHR ligand-treated mice provide strong evidence of an endogenous role of the receptor in female reproductive physiology. Deficiencies in maintaining pregnancy and surviving lactation have been reported in AHR knockout mice [45], and disruption of AHR function alters ovarian development, folliculogenesis, steroid hormone synthesis, ovulation and possibly reproductive senescence [29]. In keeping with AHR knockout mouse models, *ahr2* ^hu3335^ zebrafish are capable of producing viable embryos, but exhibit decreased reproductive success. It is important to note, however, that other ENU-induced mutations throughout the genome of this fish line could be responsible for observed phenotypic abnormalities. Zebrafish TILLING mutants require multiple outcrosses to reduce undesired mutations to background levels. Because outcrosses of the *ahr2* ^hu3335^ line were in progress at the time of this study, it is premature to attribute all phenotypic abnormalities observed in *ahr2* ^hu3335^ homozygotes to the mutation in *ahr2*. Decreased reproductive capacity of homozygous mutants, as well as fin and jaw abnormalities may represent interesting models of endogenous AHR function and certainly warrant further investigation if they persist in the mutant line following further outcrosses.

In the present study, we used TCDD as a tool to investigate AHR2 function in the *ahr2* ^hu3335^ line. We found that *ahr2* ^hu3335^ embryos were resistant to TCDD-induced developmental toxicity at concentrations that cause severe malformations in *ahr2* ^+^ embryos. *ahr2* ^hu3335^ embryos treated with 10 nM TCDD showed few signs of morbidity at 120 hpf. Transient AHR2 knockdown delays, but does not prevent, TCDD-induced mortality [25]. Therefore it would be interesting to examine longer-term effects of TCDD exposure in future experiments with the *ahr2* ^hu3335^ line.

The most well-known biomarker of AHR activation is the induction of CYP1A expression. Among the suite of cytochrome P450 metabolizing enzymes in zebrafish, CYP1A, CYP1B1, CYP1C1 and CYP1C2 are elevated in response to AHR agonist exposure [38]. In agreement with our developmental toxicity data, no elevation in CYP1A, CYP1C1 or CYP1C2 expression was observed in TCDD-exposed *ahr2* ^hu3335^ embryos. Taken together, these data support the concept that AHR2 is not functional in this line. The notable, but statistically insignificant, increase in CYP1A expression following TCDD treatment in *ahr2* ^hu3335^ embryos is likely due to TCDD activation of AHR1B, as further discussed below.

While the dependence of CYP1A activation by TCDD on AHR2 is well-established, studies with PAHs in zebrafish embryos have revealed diverse CYP1A expression patterns dependent on other AHR isoforms [46], [47]. This study represents the first time that an *in silico-*based modeling approach was utilized to investigate ligand binding by all three receptors. Molecular docking with TCDD predicted that both AHR1B and AHR2, but not AHR1A, would bind TCDD due to substitutions in the binding pocket. In contrast to TCDD, *in silico* modeling with leflunomide predicts favorable binding energies for all three zebrafish AHR isoforms. Interestingly, leflunomide docked into AHR1A with a different predicted conformation than in the other two receptors, but with equivalent affinity. This finding is particularly intriguing, as AHR1A is incapable of binding classical AHR ligands [23], is deficient in transactivation activity [21], and therefore was once considered non-functional.

We confirmed the AHR modeling results *in vivo* using CYP1A protein expression as a biomarker of AHR activation. In keeping with our mRNA expression and *in silico* modeling studies, TCDD-exposed *ahr2* ^hu3335^ larvae were largely devoid of CYP1A protein expression observed in TCDD-exposed *ahr2* ^+^ larvae. Leflunomide also induces strong vascular CYP1A protein expression in *ahr2* ^+^ larvae, but unlike with TCDD, the *ahr2* ^hu3335^ embryos exhibited striking leflunomide-induced CYP1A expression in the liver. This finding is in agreement with the modeling results. To tease apart AHR isoform-dependence of the residual CYP1A expression, we transiently knocked down the receptors individually and in combination in *ahr2* ^hu3335^ larvae. We found AHR1B-dependent vascular induction and AHR1A-dependent liver induction of CYP1A expression. Knockdown of AHR1A and AHR1B in combination prevented all CYP1A induction. Taken together, these data suggest that, contrary to previous observations with TCDD, all three AHR isoforms are involved in leflunomide-induced CYP1A expression in zebrafish larvae.

These data demonstrate that there are concrete differences in ligand binding activity of the zebrafish AHRs, and that AHR1A is not a pseudogene as previously proposed, but rather has affinity for different ligand structures. While residual CYP1A expression has been observed in TCDD-treated AHR2-morphants, it was faint and vascular in nature, attributable to incomplete knockdown [25]. Our immunohistochemical results with the *ahr2* ^hu3335^ line suggest that mild vascular expression of CYP1A is induced via AHR1B, and can be effectively knocked down to background with morpholino injection. AHR1A-dependent CYP1A expression is seemingly incongruous with previous investigation of AHR1A function *in vitro*, but the lack of a known AHR1A ligand limited previous efforts. The AHR1A-dependent CYP1A expression pattern we observed here is consistent with the reported AHR1A mRNA expression in the liver [21].

Putative AHR1A ligands could be identified with further *in silico* modeling; work by Incardona and colleagues also offers clues with several PAHs that induce CYP1A expression independently of AHR2 [46], [47], [48]. Pyrene induced liver expression of CYP1A in an AHR1A-dependent manner [47], and more recently retene-induced CYP1A expression was shown to be incompletely dependent on AHR2 [49]. Here, we offer further evidence that AHR1A is a functional receptor *in vivo*, though the transactivation requirements for this receptor remain to be elucidated. *In vitro* data with AHR chimera proteins suggest that transactivation requirements of AHR1A differ from those of AHR2 [21].

The presence of three apparently functional aryl hydrocarbon receptors in zebrafish raises several interesting questions: How do these receptors differ? What functions have led to their evolutionary conservation? And to what extent do the AHR1 receptors need to be considered in toxicological studies in zebrafish? While the presence of multiple AHRs certainly complicates study of receptor function in fish, subfunction partitioning among isoforms presents a unique opportunity to unravel the many physiological functions of the AHR that are conserved among vertebrates [20]. As summarized in Table 3, the studies presented here add to a body of research demonstrating significant differences in receptor expression, ligand binding, and mRNA induction activity. With respect to transcript localization, AHR2 is widely distributed through most organs investigated in adult zebrafish, while AHR1A is mainly expressed in the liver, and to a lesser extent in the heart, kidney and swim bladder [21]. AHR1B expression has yet to be fully characterized, but our CYP1A IHC results suggest that the isoform is widely distributed, but is expressed at much lower levels than AHR2. The subfunction partitioning of these receptors is not strictly locational. Overlapping expression of AHR2 and AHR1A has been previously described, and we also noted overlap in AHR2- and AHR1-dependent CYP1A expression patterns [23], [42]. A cell or tissue-level analysis may reveal more subtle localization differences, as has been implied in differential PAH-induced CYP1A patterns in endocardial and myocardial tissue [47], [48]. Little is yet known about the endogenous function of these receptors and their downstream transcriptional targets. If expression of AHR1A and AHR1B is limited, it may be difficult to detect significant changes in their transcriptional targets in whole embryo homogenate. As we have shown here, however, the *ahr2* ^hu3335^ line will ease the study of the other two receptors by removing the overpowering transcriptional changes induced through AHR2. The three receptors together present an intriguing opportunity to unravel multiple regulatory functions that may be conserved in the mammalian AHR.

**Table 3:** Summary of zebrafish AHR ligand binding, activity and expression.

Receptor	Receptor mRNA expression in adult zebrafish	In vitro TCDD binding and activity	Homology model predicted binding		Dominant receptor-dependent CYP1A protein induction pattern (larval)
			TCDD	Leflunomide	
**AHR1A**	heart, swimbladder, liver, kidney [21]	N [21], [23]	N	Y	liver
**AHR1B**	NA	Y [23]	Y	Y	vasculature
**AHR2**	brain, heart, muscle, swimbladder, liver, gill, skin, eye, kidney, fin [21]	Y [21], [23]	Y	Y	liver, vasculature

This is the first report of CYP1A induction dependent on all three of the zebrafish AHRs. Toxicity mediated through the AHR1 receptors, however, has not, as of yet, been documented. Pyrene-induced liver toxicity and pericardial edema were reduced with AHR1A knockdown, but AHR2 knockdown prevented the majority of the chemical's developmental effects [46], [47]. In the case of TCDD and other similarly-structured HAHs, the small binding pocket of AHR1A prevents it from having a role in ligand-induced toxicological effects. AHR1A and AHR1B receptors may hold little importance in toxicological studies with these compounds. Indeed the studies presented here support the large body of previous research indicating that TCDD-induced early life stage toxicity is mediated through AHR2. Though some CYP1A and other downstream target induction may occur via AHR1B, any developmental abnormalities caused by this pathway are more subtle than those investigated to date. The possibility remains, however, that AHR1B may play a role in later life stage impacts of TCDD. These data warrant further investigation of the AHR isoforms with structurally diverse, less-well studied compounds. Ultimately, further bioinformatic and modeling efforts with zebrafish and mammalian AHRs could help determine the best model for human AHR activity, taking into account both ligand binding and receptor expression characteristics.

This was the first time that all three AHR isoforms were knocked down in developing zebrafish. Our findings suggest that, consistent with mammalian literature, AHR function is not required to complete development [33], [50]. Without full histological evaluations of the AHR1Amo/AHR1Bmo/*ahr2* ^hu3335^ larvae at 120hpf, we cannot exclude non-lethal malformations, particularly hepatic abnormalities, which have been reported in AHR knockout mice [33], [35]. It may not be possible to fully answer the question of whether the AHR paralogues are required for hepatic development in zebrafish with the tools employed here, as the liver undergoes significant development after 72hpf, when morpholino efficacy is in decline. We therefore present the *ahr2* ^hu3335^ line as a valuable resource available to the zebrafish research community, and suggest that development of both AHR1A and AHR1B (already requested by the research community) mutant lines would further extend the power of this model for investigating both the endogenous and ligand-mediated roles of the AHR in developing vertebrates.

## Materials and Methods

### Zebrafish lines and embryos

Adult zebrafish were housed according to Institutional Animal Care and Use Committee protocols at Oregon State University on a recirculating system with water temperature of 28±1°C and a 14 h light/10 h dark schedule. Zebrafish embryos carrying a point mutation in *ahr2* (*ahr2* ^hu3335^ strain) were requested and generously provided by the Hubrecht Institute. The *ahr2* ^hu3335^ line was identified from a library of *N*-ethyl-*N*-nitrosourea (ENU)-mutagenized zebrafish using the TILLING method as previously described [51]. Offspring of heterozygous *ahr2* ^hu3335^ carriers were raised to adulthood at the Sinnhuber Aquatic Research Laboratory, and genotyped for the *ahr2* ^hu3335^ point mutation with DNA isolated from fin clips [51]. PCR amplification was performed with genomic DNA and *ahr2* gene-specific primers (Table 4), the product was purified using a QIAquick PCR purification kit (Qiagen) and sequenced with an ABI 3730 capillary sequencer at the Center for Genome Research and Biocomputing at Oregon State University. Homozygous carriers of the T to A point mutation in residue 534 (Figure 1) were identified to create an *ahr2* ^hu3335^ population. Because the TILLING method relies on random mutagenesis, mutant lines of interest carry other mutations throughout the genome. F1 fish are predicted to carry 3–6000 mutations and multiple outcrosses are necessary to reduce off-target mutations [52]. *ahr2* ^hu3335^ carriers were outcrossed to the wild type 5D (*ahr2* ^+/+^) line, and homozygous mutants were identified from an incross of their progeny. The *ahr2* ^hu3335^ mutant line has been maintained with subsequent outcrosses on the wild type 5D background, which was also used for all *ahr2* ^+^ control experiments in our laboratory.

**Table 4:** Primer sequences for PCR experiments.

Target	Forward Primer (5′- 3′)	Reverse Primer (5′- 3′)
**AHR1A**	CGCAAAAGGAGGAAACCTGTC [47]	CCTGTAGCAAAAATTCCCCCT [47]
**AHR1B**	GGTTTGTCGTCAAACAACAGTAACCACG [23]	CCACCAACACAAAGCCATTAAGAGCCTG [23]
**AHR1B-mo**	CTTTGTGTGTCGTTTCCGATGCC	GCACAGTAGAGCATATCAGCTGC
**AHR2**	TGGACTAGATCAGACAACCC	GAAGAGGGAGAGTCATTGTG
**AHR2-mut**	TATTGCTAGGCAGAGAGCAC	GATGTCTTCTGTGATGATTTCG
**CYP1A**	TGCCGATTTCATCCCTTTCC	AGAGCCGTGCTGATAGTGTC
**CYP1B1**	CTGCATTGATTTCCGAGACGTG [59]	CACACTCCGTGTTGACAGC [59]
**CYP1C1**	AGTGGCACAGTCTACTTTGAGAG [59]	TCGTCCATCAGCACTCAG [59]
**CYP1C2**	GTGGTGGAGCACAGACTAAG [59]	TTCAGTATGAGCCTCAGTCAAAC [59]
**β-ACTIN**	AAGCAGGAGTACGATGAGTC	TGGAGTCCTCAGATGCATTG

**mo**- morpholino mis-splice detection.

**mut**- mutant point mutation detection.

All developmental toxicity experiments were conducted with fertilized embryos obtained from group spawns of adult zebrafish as described previously [53]. Embryos used in experiments are defined as homozygous (*ahr2* ^hu3335^), heterozygous (*ahr2* ^hu3335/+^) or wild-type (*ahr2* ^+^) for the point mutation in AHR2.

### MicroCt imaging

Micro computed tomography (μCT) was used for nondestructive three-dimensional imaging of zebrafish heads. The fish were scanned using a Scanco μCT40 scanner (Scanco Medical AG, Basserdorf, Switzerland) at 45 kVp, 177 mA, and a voxel size of 12×12×12 mm. The heads were imaged at threshold settings of 140 (scale 0–1000).

### Homology modeling, molecular docking and binding energy calculations

Molecular modeling of zebrafish AHR2, AHR1B and AHR1A isoforms was conducted as described previously (17). Briefly, the homology models of mouse, human, rat and zebrafish AHR-LBD (ligand binding domains) were built using the NMR resolved structure of the PAS domain of human hypoxia-inducible-factor 2α as the 3D-template. Models were then refined in the internal coordinates with Molsoft ICM v3.5-1p. Molecular docking of TCDD and leflunomide ligands and binding energy calculation were performed as reported [17].

### Chemical exposures and developmental toxicity assessment

TCDD (99.2% purity in DMSO, Cambridge Isotope Laboratories) and leflunomide (Sigma-Aldrich) were dissolved in DMSO. All exposures were conducted in E2 embryo medium with staged embryos [54]. Embryos were batch exposed to 0.1, 1, 10 nM TCDD or 0.1% DMSO vehicle control in 2 mL embryo medium in glass vials from 6–24 hours post fertilization (hpf). Embryos were then rinsed 4× with embryo media and transferred to plastic dishes to develop until the indicated experimental time points. Embryo homogenate for mRNA expression analysis was collected at 48 hpf, and developmental toxicity of TCDD exposure was assessed by visually inspecting embryos at 120 hpf for malformations as previously described [55] with three biological replicates. Developmental toxicity assay data were analyzed by fitting a 2 parameter logistic regression model to the concentration-response data for each malformation. Significance of the TCDD concentration-response curve was calculated for each fish line. Differential responses were assessed with a t-test to compare the parameters from the *ahr2* ^+^ model to those from the *ahr2* ^hu3335^ model. No adjustment for multiplicity was made. R software v12.0 [56] was used for these analyses.

For leflunomide exposures, embryos were transferred into individual wells of a 96-well plate and exposed to 10 µM leflunomide or 0.1% DMSO control in 100 µl embryo medium from 48–72 hpf, when they were humanely euthanized and fixed for immunohistochemistry analysis.

### Total RNA isolation and reverse transcription

For qRT-PCR studies, 20 embryos per treatment group were homogenized in TRIzol (Invitrogen) and stored at −80°C until use. Total RNA was isolated via phenol/guanidine isothiocyanate/chloroform separation. For morpholino splice-blocking confirmation, 15 embryos were homogenized in RNAzol (Molecular Research Center) for total RNA isolation. RNA was quantified using a SynergyMx microplate reader (Biotek) with the Gen5 Take3 module to calculate 260/280 O.D. ratios. Superscript III First-Strand Synthesis (Invitrogen) was used with oligo(dT) primer to reverse transcribe cDNA from total RNA.

### Quantitative RT-PCR

Relative abundance of AHR1A, AHR1B, AHR2, CYP1A, CYP1B1, CYP1C1 and CYP1C2 mRNA transcripts were assessed in whole embryo homogenate. Gene-specific primers (MWG Operon) are listed in Table 4. All qRT-PCR assays were performed in 20 µl reactions consisting of 10 µl Power SYBR Green PCR master mix (Applied Biosystems), 0.4 ul each primer, 9.2 ul H_2_O and 50 ng equivalents of cDNA. Amplification (Step One Plus, Applied Biosystems) was performed with cycling parameters as follows: 95°C for 10 min; 40 cycles of 95°C for 15 s, 60°C for 1 min; 95°C for 15 sec and 60°C for 1 min. A melt curve was performed at 3° increments to assess for multiple products.

qRT-PCR analysis was performed with StepOne Software v2.1 (Applied Biosystems) using the ΔΔCt method with genes of interest normalized to β-actin [57]. Three independent biological replicates were assessed and statistically analyzed by comparing *ahr2* ^hu3335^ to *ahr2* ^+^ or TCDD-treated to control with a Student's t-test using Graphpad Prism 5.01 software (Graphpad Software Inc. La Jolla, CA).

### Morpholino injection

Splice-blocking morpholinos designed against AHR1A and AHR1B were purchased from Gene Tools (Philomath, OR). The AHR1A splice-blocking morpholino (AHR1Amo, 5′ CTTTTGAAGTGACTTTTGGCCCGCA 3′) was described previously [47] and was tagged on the 3′ end with fluorescein. We designed a morpholino to target the exon7/intron7 boundary of AHR1B (AHR1Bmo, 5′ ACACAGTCGTCCATGATTACTTTGC 3′). A standard control morpholino from Gene Tools (cmo, 5′ CCTCTTACCTCAGTTACAATTTATA 3′) was used as a negative control. *ahr2* ^hu3335^ embryos were injected at the 1–2 cell stage with approximately 2 nl of 1.5 mM morpholino dissolved in ultrapure water with 0.5% phenol red. For AHR1Amo +AHR1Bmo co-injections, the final concentration of each morpholino was 0.83 mM. Embryos were allowed to develop in fish water and screened for successful morpholino incorporation with fluorescein visualization at 24 hpf. mRNA mis-splice was confirmed with PCR primers flanking the target sites at 24 and 72 hpf (AHR1A and AHR1B-mo primers Table 4).

### Immunohistochemistry (IHC)

Wild-type strain 5D and *ahr2* ^hu3335^ embryos treated with 1 nM TCDD (or 0.1% DMSO control) from 6–24 hpf were fixed at 120 hpf in 4% paraformaldehyde (J.T. Baker) overnight at 4°C. Leflunomide treated embryos (48–72 hpf) were fixed at 72 hpf to capture the window of morpholino efficacy. Mouse α fish CYP1A monoclonal (1∶500 dilution, Biosense laboratories) and goat α human HNF4α polyclonal (1∶100 dilution, Santa Cruz Biotechnology) primary antibodies were used. Secondary antibodies consisted of Alexafluor® 546 rabbit α mouse IgG (H+L) (1∶1000) and Alexafluor® 488 donkey α goat IgG (H+L) (1∶1000) (Molecular Probes, Eugene, OR). Immunohistochemistry was performed as previously described [27]. Briefly, whole fixed embryos were permeabilized with 0.005% trypsin on ice for 10 min, washed 3× with PBST and post-fixed in 4% paraformaldehyde for 10 min. Samples were blocked for 1 h in 10% normal goat serum (single labeling) or BlockAid (double labeling) (Invitrogen). Samples were incubated with primary antibodies overnight at 4°C, followed by 4 30 min washes in PBST and incubation with secondary antibody overnight at 4°C. At least 8 embryos per treatment group were imaged by epi-fluorescence microscopy using a Zeiss Axiovert 200 M microscope with 5× and 10× objectives.

## Supporting Information

Figure S1 **Confirmation of morpholino target mis-splice.** PCR amplification of AHR1A and AHR1B fragments spanning the morpholino target sites were performed with mRNA isolated from 72 hpf whole embryo homogenate. Lane 1: control morpholino (cmo) injected, Lane 2: AHR1A+AHR1Bmo injected, Lane 3: AHR1Bmo injected. WT: wild-type, INS: insertion, DEL: deletion.
(TIF)

## References

[1] Fukunaga BN, Probst MR, Reisz-Porszasz S, Hankinson O (1995). Identification of functional domains of the aryl hydrocarbon receptor.. J Biol Chem.

[2] Fukunaga BN, Hankinson O (1996). Identification of a novel domain in the aryl hydrocarbon receptor required for DNA binding.. J Biol Chem.

[3] Denison MS, Nagy SR (2003). Activation of the aryl hydrocarbon receptor by structurally diverse exogenous and endogenous chemicals.. Annu Rev Pharmacol Toxicol.

[4] Nebert DW, Robinson JR, Niwa A, Kumaki K, Poland AP (1975). Genetic expression of aryl hydrocarbon hydroxylase activity in the mouse.. J Cell Physiol.

[5] Schmidt JV, Bradfield CA (1996). Ah receptor signaling pathways.. Annu Rev Cell Dev Biol.

[6] Nebert DW, Roe AL, Dieter MZ, Solis WA, Yang Y (2000). Role of the aromatic hydrocarbon receptor and [Ah] gene battery in the oxidative stress response, cell cycle control, and apoptosis.. Biochem Pharmacol.

[7] Sartor MA, Schnekenburger M, Marlowe JL, Reichard JF, Wang Y (2009). Genomewide analysis of aryl hydrocarbon receptor binding targets reveals an extensive array of gene clusters that control morphogenetic and developmental programs.. Environ Health Perspect.

[8] Nebert DW, Dalton TP, Okey AB, Gonzalez FJ (2004). Role of aryl hydrocarbon receptor-mediated induction of the CYP1 enzymes in environmental toxicity and cancer.. J Biol Chem.

[9] Gu YZ, Hogenesch JB, Bradfield CA (2000). The PAS superfamily: sensors of environmental and developmental signals.. Annu Rev Pharmacol Toxicol.

[10] Kerkvliet NI (2009). AHR-mediated immunomodulation: the role of altered gene transcription.. Biochem Pharmacol.

[11] Puga A, Ma C, Marlowe JL (2009). The aryl hydrocarbon receptor cross-talks with multiple signal transduction pathways.. Biochem Pharmacol.

[12] Hahn ME (2002). Aryl hydrocarbon receptors: diversity and evolution.. Chem Biol Interact.

[13] Ema M, Ohe N, Suzuki M, Mimura J, Sogawa K (1994). Dioxin binding activities of polymorphic forms of mouse and human arylhydrocarbon receptors.. J Biol Chem.

[14] Wirgin I, Roy NK, Loftus M, Chambers RC, Franks DG Mechanistic basis of resistance to PCBs in Atlantic tomcod from the Hudson River.. Science.

[15] Hahn ME, Karchner SI, Evans BR, Franks DG, Merson RR (2006). Unexpected diversity of aryl hydrocarbon receptors in non-mammalian vertebrates: insights from comparative genomics.. J Exp Zool A Comp Exp Biol.

[16] Murray IA, Flaveny CA, Chiaro CR, Sharma AK, Tanos RS (2011). Suppression of cytokine-mediated complement factor gene expression through selective activation of the Ah receptor with 3′,4′-dimethoxy-alpha-naphthoflavone.. Mol Pharmacol.

[17] Bisson WH, Koch DC, O'Donnell EF, Khalil SM, Kerkvliet NI (2009). Modeling of the aryl hydrocarbon receptor (AhR) ligand binding domain and its utility in virtual ligand screening to predict new AhR ligands.. J Med Chem.

[18] Billiard SM, Hahn ME, Franks DG, Peterson RE, Bols NC (2002). Binding of polycyclic aromatic hydrocarbons (PAHs) to teleost aryl hydrocarbon receptors (AHRs).. Comp Biochem Physiol B Biochem Mol Biol.

[19] Carney SA, Prasch AL, Heideman W, Peterson RE (2006). Understanding dioxin developmental toxicity using the zebrafish model.. Birth Defects Res A Clin Mol Teratol.

[20] Postlethwait J, Amores A, Cresko W, Singer A, Yan YL (2004). Subfunction partitioning, the teleost radiation and the annotation of the human genome.. Trends Genet.

[21] Andreasen EA, Hahn ME, Heideman W, Peterson RE, Tanguay RL (2002). The zebrafish (Danio rerio) aryl hydrocarbon receptor type 1 is a novel vertebrate receptor.. Mol Pharmacol.

[22] Tanguay RL, Abnet CC, Heideman W, Peterson RE (1999). Cloning and characterization of the zebrafish (Danio rerio) aryl hydrocarbon receptor.. Biochim Biophys Acta.

[23] Karchner SI, Franks DG, Hahn ME (2005). AHR1B, a new functional aryl hydrocarbon receptor in zebrafish: tandem arrangement of ahr1b and ahr2 genes.. Biochem J.

[24] Antkiewicz DS, Peterson RE, Heideman W (2006). Blocking expression of AHR2 and ARNT1 in zebrafish larvae protects against cardiac toxicity of 2,3,7,8-tetrachlorodibenzo-p-dioxin.. Toxicol Sci.

[25] Prasch AL, Teraoka H, Carney SA, Dong W, Hiraga T (2003). Aryl hydrocarbon receptor 2 mediates 2,3,7,8-tetrachlorodibenzo-p-dioxin developmental toxicity in zebrafish.. Toxicol Sci.

[26] Teraoka H, Dong W, Tsujimoto Y, Iwasa H, Endoh D (2003). Induction of cytochrome P450 1A is required for circulation failure and edema by 2,3,7,8-tetrachlorodibenzo-p-dioxin in zebrafish.. Biochem Biophys Res Commun.

[27] Mathew LK, Andreasen EA, Tanguay RL (2006). Aryl hydrocarbon receptor activation inhibits regenerative growth.. Mol Pharmacol.

[28] Billiard SM, Timme-Laragy AR, Wassenberg DM, Cockman C, Di Giulio RT (2006). The role of the aryl hydrocarbon receptor pathway in mediating synergistic developmental toxicity of polycyclic aromatic hydrocarbons to zebrafish.. Toxicol Sci.

[29] Hernandez-Ochoa I, Karman BN, Flaws JA (2009). The role of the aryl hydrocarbon receptor in the female reproductive system.. Biochem Pharmacol.

[30] Singh KP, Casado FL, Opanashuk LA, Gasiewicz TA (2009). The aryl hydrocarbon receptor has a normal function in the regulation of hematopoietic and other stem/progenitor cell populations.. Biochem Pharmacol.

[31] Matsumura F, Puga A, Tohyama C (2009). Biological functions of the arylhydrocarbon receptor: beyond induction of cytochrome P450s. Introduction to this special issue.. Biochem Pharmacol.

[32] Peterson RE, Theobald HM, Kimmel GL (1993). Developmental and reproductive toxicity of dioxins and related compounds: cross-species comparisons.. Crit Rev Toxicol.

[33] Schmidt JV, Su GH, Reddy JK, Simon MC, Bradfield CA (1996). Characterization of a murine Ahr null allele: involvement of the Ah receptor in hepatic growth and development.. Proc Natl Acad Sci U S A.

[34] Fernandez-Salguero P, Pineau T, Hilbert DM, McPhail T, Lee SS (1995). Immune system impairment and hepatic fibrosis in mice lacking the dioxin-binding Ah receptor.. Science.

[35] Lahvis GP, Pyzalski RW, Glover E, Pitot HC, McElwee MK (2005). The aryl hydrocarbon receptor is required for developmental closure of the ductus venosus in the neonatal mouse.. Mol Pharmacol.

[36] Wittkopp N, Huntzinger E, Weiler C, Sauliere J, Schmidt S (2009). Nonsense-mediated mRNA decay effectors are essential for zebrafish embryonic development and survival.. Mol Cell Biol.

[37] Cubbage CC, Mabee PM (1996). Development of the cranium and paired fins in the zebrafish Danio rerio (Ostariophysi, cyprinidae).. Journal of Morphology.

[38] Jonsson ME, Jenny MJ, Woodin BR, Hahn ME, Stegeman JJ (2007). Role of AHR2 in the expression of novel cytochrome P450 1 family genes, cell cycle genes, and morphological defects in developing zebra fish exposed to 3,3′,4,4′,5-pentachlorobiphenyl or 2,3,7,8-tetrachlorodibenzo-p-dioxin.. Toxicol Sci.

[39] O'Donnell EF, Saili KS, Koch DC, Kopparapu PR, Farrer D (2010). The anti-inflammatory drug leflunomide is an agonist of the aryl hydrocarbon receptor.. PLoS One.

[40] Pandini A, Denison MS, Song Y, Soshilov AA, Bonati L (2007). Structural and functional characterization of the aryl hydrocarbon receptor ligand binding domain by homology modeling and mutational analysis.. Biochemistry.

[41] Pandini A, Soshilov AA, Song Y, Zhao J, Bonati L (2009). Detection of the TCDD binding-fingerprint within the Ah receptor ligand binding domain by structurally driven mutagenesis and functional analysis.. Biochemistry.

[42] Andreasen EA, Spitsbergen JM, Tanguay RL, Stegeman JJ, Heideman W (2002). Tissue-specific expression of AHR2, ARNT2, and CYP1A in zebrafish embryos and larvae: effects of developmental stage and 2,3,7,8-tetrachlorodibenzo-p-dioxin exposure.. Toxicol Sci.

[43] Carney SA, Peterson RE, Heideman W (2004). 2,3,7,8-Tetrachlorodibenzo-p-dioxin activation of the aryl hydrocarbon receptor/aryl hydrocarbon receptor nuclear translocator pathway causes developmental toxicity through a CYP1A-independent mechanism in zebrafish.. Mol Pharmacol.

[44] Dong PD, Munson CA, Norton W, Crosnier C, Pan X (2007). Fgf10 regulates hepatopancreatic ductal system patterning and differentiation.. Nat Genet.

[45] Abbott BD, Schmid JE, Pitt JA, Buckalew AR, Wood CR (1999). Adverse reproductive outcomes in the transgenic Ah receptor-deficient mouse.. Toxicol Appl Pharmacol.

[46] Incardona JP, Carls MG, Teraoka H, Sloan CA, Collier TK (2005). Aryl hydrocarbon receptor-independent toxicity of weathered crude oil during fish development.. Environ Health Perspect.

[47] Incardona JP, Day HL, Collier TK, Scholz NL (2006). Developmental toxicity of 4-ring polycyclic aromatic hydrocarbons in zebrafish is differentially dependent on AH receptor isoforms and hepatic cytochrome P4501A metabolism.. Toxicol Appl Pharmacol.

[48] Incardona JP, Linbo TL, Scholz NL. Cardiac toxicity of 5-ring polycyclic aromatic hydrocarbons is differentially dependent on the aryl hydrocarbon receptor 2 isoform during zebrafish development.. Toxicol Appl Pharmacol.

[49] Scott JA, Incardona JP, Pelkki K, Shepardson S, Hodson PV (2011). AhR2-mediated, CYP1A-independent cardiovascular toxicity in zebrafish (Danio rerio) embryos exposed to retene.. Aquat Toxicol.

[50] Gonzalez FJ, Fernandez-Salguero P (1998). The aryl hydrocarbon receptor: studies using the AHR-null mice.. Drug Metab Dispos.

[51] Wienholds E, van Eeden F, Kosters M, Mudde J, Plasterk RH (2003). Efficient target-selected mutagenesis in zebrafish.. Genome Res.

[52] Moens CB, Donn TM, Wolf-Saxon ER, Ma TP (2008). Reverse genetics in zebrafish by TILLING.. Brief Funct Genomic Proteomic.

[53] Reimers MJ, La Du JK, Periera CB, Giovanini J, Tanguay RL (2006). Ethanol-dependent toxicity in zebrafish is partially attenuated by antioxidants.. Neurotoxicol Teratol.

[54] Kimmel CB, Ballard WW, Kimmel SR, Ullmann B, Schilling TF (1995). Stages of embryonic development of the zebrafish.. Dev Dyn.

[55] Truong L, Harper SL, Tanguay RL (2011). Evaluation of embryotoxicity using the zebrafish model.. Methods Mol Biol.

[56] Team RDC, editor (2010). R: A language and environment for statistical computing..

[57] Livak KJ, Schmittgen TD (2001). Analysis of relative gene expression data using real-time quantitative PCR and the 2(-Delta Delta C(T)) Method.. Methods.

[58] Hahn ME (1998). The aryl hydrocarbon receptor: a comparative perspective.. Comp Biochem Physiol C Pharmacol Toxicol Endocrinol.

[59] Jonsson ME, Franks DG, Woodin BR, Jenny MJ, Garrick RA (2009). The tryptophan photoproduct 6-formylindolo[3,2-b]carbazole (FICZ) binds multiple AHRs and induces multiple CYP1 genes via AHR2 in zebrafish.. Chem Biol Interact.

