# Supplementary figures and images for: AHR2 Mutant Reveals Functional Diversity of Aryl Hydrocarbon Receptors in Zebrafish
Source: PLoS One. 2012 Jan 5;7(1):e29346. doi: 10.1371/journal.pone.0029346 (PMC3252317; doi:10.1371/journal.pone.0029346)

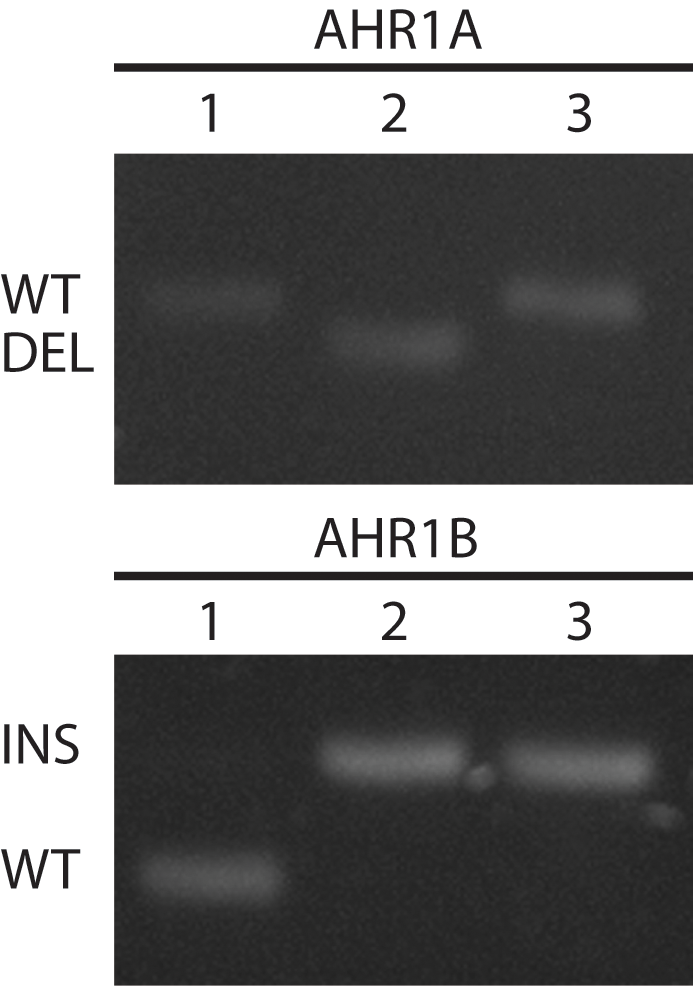

Supplement: Figure S1 — Confirmation of morpholino target mis-splice. PCR amplification of AHR1A and AHR1B fragments spanning the morpholino target sites were performed with mRNA isolated from 72 hpf whole embryo homogenate. Lane 1: control morpholino (cmo) injected, Lane 2: AHR1A+AHR1Bmo injected, Lane 3: AHR1Bmo injected. WT: wild-type, INS: insertion, DEL: deletion. (TIF) [file pone.0029346.s001.tif]
